# Comparative Transcriptome Analysis Unravels Defense Pathways of *Fraxinus velutina* Torr Against Salt Stress

**DOI:** 10.3389/fpls.2022.842726

**Published:** 2022-03-04

**Authors:** Xinmei Ma, Jian Ning Liu, Liping Yan, Qiang Liang, Hongcheng Fang, Changxi Wang, Yuhui Dong, Zejia Chai, Rui Zhou, Yan Bao, Wenrui Hou, Ke Qiang Yang, Dejun Wu

**Affiliations:** ^1^College of Forestry, Shandong Agricultural University, Tai’an, China; ^2^Shandong Provincial Academy of Forestry, Jinan, China; ^3^State Forestry and Grassland Administration Key Laboratory of Silviculture in the Downstream Areas of the Yellow River, Shandong Agricultural University, Tai’an, China; ^4^Shandong Taishan Forest Ecosystem Research Station, Shandong Agricultural University, Tai’an, China

**Keywords:** *Fraxinus velutina* Torr, salt stress, comparative transcriptome, stress-responsive gene, defense response

## Abstract

*Fraxinus velutina* Torr with high salt tolerance has been widely grown in saline lands in the Yellow River Delta, China. However, the salt-tolerant mechanisms of *F. velutina* remain largely elusive. Here, we identified two contrasting cutting clones of *F. velutina*, R7 (salt-tolerant), and S4 (salt-sensitive) by measuring chlorophyll fluorescence characteristics (Fv/Fm ratio) in the excised leaves and physiological indexes in roots or leaves under salt treatment. To further explore the salt resistance mechanisms, we compared the transcriptomes of R7 and S4 from leaf and root tissues exposed to salt stress. The results showed that when the excised leaves of S4 and R7 were, respectively, exposed to 250 mM NaCl for 48 h, *Fv*/*Fm* ratio decreased significantly in S4 compared with R7, confirming that R7 is more tolerant to salt stress. Comparative transcriptome analysis showed that salt stress induced the significant upregulation of stress-responsive genes in R7, making important contributions to the high salt tolerance. Specifically, in the R7 leaves, salt stress markedly upregulated key genes involved in plant hormone signaling and mitogen-activated protein kinase signaling pathways; in the R7 roots, salt stress induced the upregulation of main genes involved in proline biosynthesis and starch and sucrose metabolism. In addition, 12 genes encoding antioxidant enzyme peroxidase were all significantly upregulated in both leaves and roots. Collectively, our findings revealed the crucial defense pathways underlying high salt tolerance of R7 through significant upregulation of some key genes involving metabolism and hub signaling pathways, thus providing novel insights into salt-tolerant *F. velutina* breeding.

## Introduction

Soil salinization has become a global major challenge. Saline soils are a major contributor to the regional fragile ecosystems, thereby posing a heavy burden on the sustainable advancement of local economies (Egamberdieva et al., 2019; Wani et al., 2020). As one kind of the most important tree species for afforestation, the *Fraxinus* species play crucial roles in ecological restoration (Yildiz et al., 2018; He et al., 2021). Thus, it is of importance to breeding high salt-tolerant *Fraxinus* species suitable for afforestation, especially in the areas with heavy saline-alkaline pollution. *Fraxinus velutina* Torr is a deciduous tree native to southwestern North America. Due to the rapid growth rate and superior salinity tolerance, the species has been intentionally introduced and widely planted in saline land in the Yellow River Delta, China (Mao et al., 2017). However, the molecular mechanisms underlying the high salt tolerance of *F. velutina* remain largely unknown.

Saline stress imposes primary stresses (ionic and osmotic) and secondary stresses (oxidative stress) on plants (Zhu, 2002; Yang and Guo, 2018a). Thus, plants mainly rely on the reestablishment of cellular homeostasis, namely, ionic, osmotic, and reactive oxygen species (ROS) to cope with salt stress. The process is complicated and involved in multiple signals and pathways, such as Ca^2+^, plant hormone, and ROS signaling pathways (Yang and Guo, 2018b). After salt exposure, the excessive extracellular Na^+^ induces the generation of several second messengers (e.g., cytosolic Ca^2+^), which are sensed by their sensors/receptors and protein kinases including mitogen-activated protein kinase (MAPK) and then transduce the stimuli signals into downstream components that switch on transcriptional cascades to defense against salt stress (Chinnusamy et al., 2004; Huang et al., 2012; Van Zelm et al., 2020; Chen et al., 2021). Plant hormones, namely, ethylene, salicylic acid, and abscisic acid (ABA) play irreplaceable roles in the defense response of plants to salt stress (Bari and Jones, 2009; Yu et al., 2020). For instance, salt stress can enhance ABA signaling and activate an ABA-dependent responsive complex to cope with saline stress in plants (Umezawa et al., 2009; Cai et al., 2017; Soma et al., 2017; Lin et al., 2021). In addition, salt stress induces the rapid production of ROS, which is sensed by ROS sensor/receptor and then transduced to regulate the defense response of plants under salt stress (Ashraf, 2009; Yang and Guo, 2018b). However, different plants or even different accessions of the same plant species respond differently to salt stress (Seki et al., 2003; Ji et al., 2013). Therefore, an in-depth understanding of the defense response of *F. velutina* against salt stress is an essential aid to breeding work on salt resistance.

Transcriptome profiling has been widely used to analyze salt-induced gene expression in various plants (Zhu et al., 2019; Zhang et al., 2020; Li et al., 2021; Ma et al., 2021). With the aid of RNA sequencing, significant pathways of differentially expressed genes (DEGs) are identified that would otherwise have been overlooked. In this study, two contrasting materials, *F. velutina* R7 (salt-tolerant) and S4 (salt-sensitive) were identified by measuring chlorophyll fluorescence characteristics (*Fv*/*Fm* ratio) in the excised leaves and physiological indexes in roots or leaves under salt treatment. To further explore the mechanisms underlying salt tolerance, a comparative transcriptome analysis was performed on the leaf and root of R7 and S4 clone exposed to salt stress. Our findings revealed the crucial defense response genes underlying high salt tolerance, thus providing insights into the salt-tolerant *F. velutina* breeding.

## Materials and Methods

### Plant Materials

The *F. velutina* materials were obtained from the Experimental Base of Afforestation on Saline—Alkali Soil of Shandong Provincial Academy of Forestry, Shouguang City, Shandong Province, China (118°42′9.18″ E, 37°9′38.94″ N), China. The salt-tolerant *F. velutina* R7 accession and salt-sensitive *F. velutina* S4 accession were identified from 189 *F. velutina* accessions (unpublished data) by measuring *Fv*/*Fm* ratio on the excised leaves exposed to 250 mM NaCl for 48 h according to the previously described method (Smethurst et al., 2009). Due to easy availability and appropriate size of 1-year-old cuttings of *F. velutina* (height: 26.40 ± 0.50 cm) for the experiment. In addition, 1-year-old cuttings are frequently used as plant materials for salt resistance research (Gucci et al., 1997; Tsabarducas et al., 2015; Ran et al., 2021). So, 1-year-old cuttings of *F. velutina* R7 and S4 were used in this study. After gently removing the soil around the root, the cuttings were firstly precultivated in distilled water for 2 weeks and then transferred to a plastic container containing 6 L of half-strength Hoagland’s solution for 4 weeks. The solutions were refreshed every 7 days. The cuttings were grown in a growth incubator (LICHEN, Shanghai, China) under 25/20°C (day/night temperature), 65% relative humidity, 16 h light (1,200 μmol m^–2^ s^–1^)/8 h dark.

### Chlorophyll Fluorescence

As previously described (Gabilly et al., 2019), chlorophyll fluorescence parameters (*Fv*/*Fm* ratio) were determined using a pulse-amplitude modulated chlorophyll fluorometer (FMS2, Hansatech Instruments, Pentney King’s Lynn, United Kingdom). The images reflecting chlorophyll fluorescence parameters were acquired with FluorCam imaging fluorimeters (Photon Systems Instruments, Brno, Czech Republic) according to the instructions of the manufacturer.

### Salt Treatment

After 6 weeks of acclimatization, the healthy cuttings with appropriate size (height: 26.40 ± 0.50 cm) for this experiment were selected and exposed to the solution containing 250 mM NaCl for 12 h, and the clone without NaCl treatment were considered as control. The NaCl concentration and exposure time were selected based on our preliminary tests and previous studies (Li et al., 2012; Yan et al., 2019; Catalá et al., 2021). The leaves and roots of each clone from three independent biological replicates were harvested and stored in -80°C until being used.

For easy understanding, the letters “S” and “C” were used to represent the salt-treated samples and control, respectively, while “L” and “R” represented the leaves and roots samples, respectively. For instance, the sample R7SL represented the leaves of R7 clone treated with NaCl solution.

### Physiological Parameter Measurements

The contents of proline, soluble sugar, and H_2_O_2_ were determined using the commercial kits purchased from Nanjing Jiangcheng Bioengineering Institute (Nanjing, China) following the instructions of the manufacturer. Histochemical location of H_2_O_2_ was conducted by staining with 3,3′-diaminobenzidine (DAB) according to the previously described (Daudi and O’Brien, 2012). Cell death was determined by trypan blue staining as previously described (Pogány et al., 2009). Each sample group contained five biological replicates.

### Transcriptome Sequencing

Total RNA was extracted using the GeneJET Plant RNA Purification Mini Kit (Thermo Fisher Scientific, Waltham, Massachusetts, United States). The concentration and integrity of RNA were evaluated using a NanoDrop ND-2000 (Thermo Fisher Scientific, Waltham, Massachusetts, United States) and Agilent Bioanalyzer 2100 (Agilent Technologies, Santa Clara, California, United States), respectively. About 1 μg of qualified total RNA was used to construct cDNA libraries with an insert size of 350 bp using the TruSeq RNA Sample Preparation Kit v2 (Illumina, San Diego, California, United States). The qualified libraries were sequenced on an Illumina NovaSeq 6000 platform (KeGene Science and Technology Corporation Ltd., Shandong, China) with a paired-end 150 mode.

### Transcriptome Data Analysis

The raw reads obtained were trimmed using Trimmomatic v0.39 (Bolger et al., 2014), and the high-quality reads obtained were mapped to velvet ash reference genome^1^ (Kelly et al., 2020) using HISAT2 version 2.2.1 software (Kim et al., 2015). The transcripts were assembled and quantified using StringTie v2.1.5 (Pertea et al., 2015), and the gene expression levels were measured using fragments per kilobase of transcript per million fragments mapped (FPKM). The genes with FPKM ≥ 5 in at least one sample were selected to perform differential gene expression analysis using DESeq2 v4.1 (Love et al., 2014) based on | log2 (fold change)| ≥ 2 and false discovery rate < 0.01.

### Gene Set Enrichment Analysis

The Gene Ontology (GO) enrichment analysis of the DEGs was performed using the Cytoscape v3.9.0 plug-in ClueGO v2.5.8 (Bindea et al., 2009). Kyoto Encyclopedia of Genes and Genomes (KEGG) pathway enrichment analysis of the DEGs was carried out using clusterProfiler v4.2.0 (Yu et al., 2012). The transcription factors were identified by aligning all the transcripts obtained against the plant TF database PlantTFDB v5.0 (Tian et al., 2020).

### Quantitative Real-Time Reverse Transcription PCR Analysis

To validate the reliability of transcriptome sequencing data, 16 DEGs were randomly selected to perform quantitative real-time (qRT)-PCR analysis. The CFX Connect Real-Time System (Bio-Rad, Hercules, CA, United States) was used for qRT-PCR. The PCR assays were conducted as previously described (Fang et al., 2021). The actin genes were chosen as internal reference (Li et al., 2013, 2019). The genes were quantified using the 2^–ΔΔCT^ method (Arocho et al., 2006). Each sample group contained three biological replicates. The primers are listed in Supplementary Table 1.

### Statistical Analysis

Statistical data were represented as mean ± SD. The Student’s *t*-test was used to determine the differences between the two groups. The statistical analyses were performed with GraphPad Prism v9.0 (GraphPad Software Inc., La Jolla, United States). *P < 0.05, ^**^P < 0.01, and ^***^P < 0.001 represented statistical significance.

## Results

### *Fraxinus velutina* R7 Accession Is More Salt Tolerant Than S4

**FIGURE 1:**
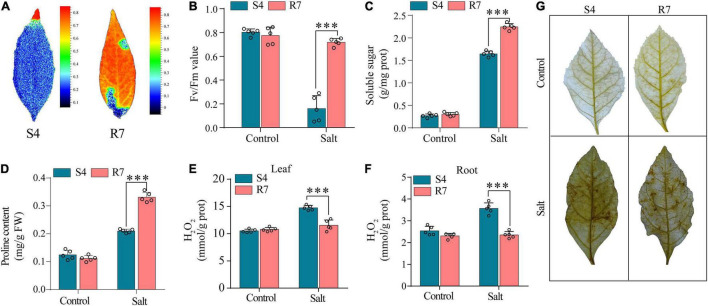
Determination of selected biochemical parameters in *F. velutina* R7 and S4. **(A)** The images reflecting chlorophyll fluorescence parameters. **(B)** Chlorophyll fluorescence parameters (*Fv*/*Fm* ratio) of the excised leaves of *F. velutina* R7 and S4 exposed to 250 mM NaCl for 48 h. Soluble sugar **(C,D)** proline content in R7 and S4 roots with or without salt treatment. **(E,F)** H_2_O_2_ content in leaves **(E)** and roots **(F)** of R7 and S4 before and after salt stress. **(G)** *In situ* visualization of H_2_O_2_ accumulation by DAB staining in R7 and S4 leaves before and after salt stress. Error bars represented SD (*n* = 5). ^***^P < 0.001 represented significant differences.

To evaluate the salt tolerance between R7 and S4, the *Fv*/*Fm* parameters in the salt-treated excised leaves were determined. The results showed that when the excised leaves of S4 and R7 were, respectively, exposed to 250 mM NaCl for 48 h, *Fv*/*Fm* ratio decreased significantly in S4 than in R7 (Figures 1A,B), suggesting that R7 is more salt tolerant.

Several selected biochemical parameters, such as soluble sugars and proline content in roots, and H_2_O_2_ content (both leaves and roots) of R7 and S4 clone exposed to salt stress were determined. The results showed that the levels of soluble sugars and proline in R7 roots were significantly higher than in S4 (Figures 1C,D). Moreover, R7 leaves and roots presented lower levels of H_2_O_2_ than S4 under salt stress (Figures 1E,F), which was further confirmed by the histochemical staining (Figure 1G). The above results further demonstrated that R7 is more tolerant to salt stress.

### Transcriptome Sequencing of R7 and S4 Accessions

To explore the differences between R7 and S4 accessions regarding gene expressions, a comparative transcriptome analysis was performed on the leaf and root of R7 and S4 clone exposed to salt stress. The results showed that 1.01 billion clean reads were obtained, with an average Q30 value was 98%. Then the clean reads were mapped to the *F. velutina* genome assembly and the results showed that 73.96–94.85% of total reads were mapped to the genome. After transcript assembly, 32,887 genes were finally produced by RNA sequencing (Supplementary Table 3).

To evaluate the consistency among the biological replicates, hierarchical clustering of all samples based on the correlation coefficient γ^2^ between each sample was performed. The results showed that leaf and root samples were clustered individually whereas the three biological replicates of each group were clustered together (Supplementary Figure 1), indicating that the biological replicates in each group are highly consistent.

**FIGURE 2:**
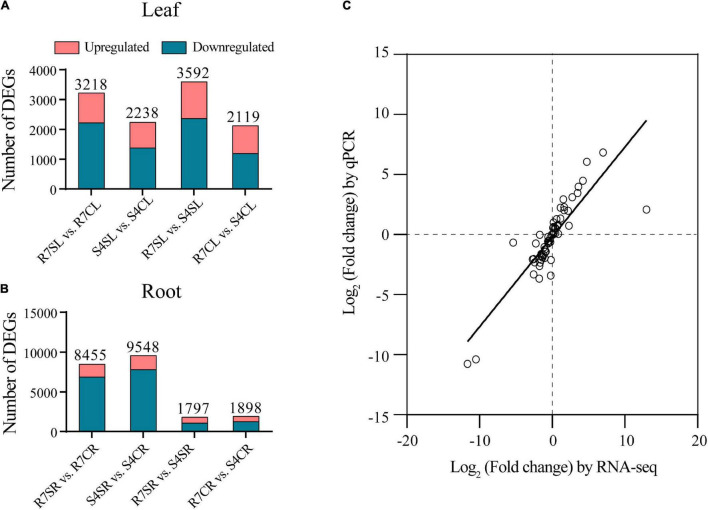
Transcriptome data and the differentially expressed genes (DEGs) in *F. velutina* R7 and S4 plants with or without salt treatment. **(A)** The DEG number of each pairwise comparison in leaf samples. **(B)** The DEG number of each pairwise comparison in root samples. **(C)** Correlation between qPCR (*X*-axis) and RNA-seq (*Y*-axis) was calculated based on log2 (fold change).

To identify salt-responsive genes, the differential gene expression analysis of pairwise comparisons R7SL vs. R7CL, S4SL vs. S4CL, R7SL vs. S4SL, and R7CL vs. S4CL in leaves and R7SR vs. R7CR, S4SR vs. S4CR, R7SR vs. S4SR, and R7CR vs. S4CR in roots were performed, respectively (Supplementary Table 4). The results showed that 3,218, 2,238, 3,592, and 2,119 DEGs were identified in comparisons R7SL vs. R7CL, S4SL vs. S4CL, R7SL vs. S4SL, and R7CL vs. S4CL in leaves, respectively (Figure 2A and Supplementary Figure 2). There were 8,455, 9,548, 1,797, and 1,898 DEGs in comparisons R7SR vs. R7CR, S4SR vs. S4CR, R7SR vs. S4SR, and R7CR vs. S4CR in roots, respectively (Figure 2B and Supplementary Figure 3).

To validate the reliability of RNA sequencing data, 16 DEGs were randomly selected to perform qRT-PCR analysis. The results showed a high correlation coefficient (*R*^2^ = 0.7266) between RNA sequencing data and qRT-PCR results, indicating that the RNA sequencing data are reliable (Figure 2C). We, therefore, concluded that the global transcriptome in R7 is altered than in S4.

### *Fraxinus velutina* R7 Enriched Stress-Responsive Genes Under Salt Stress

**FIGURE 3:**
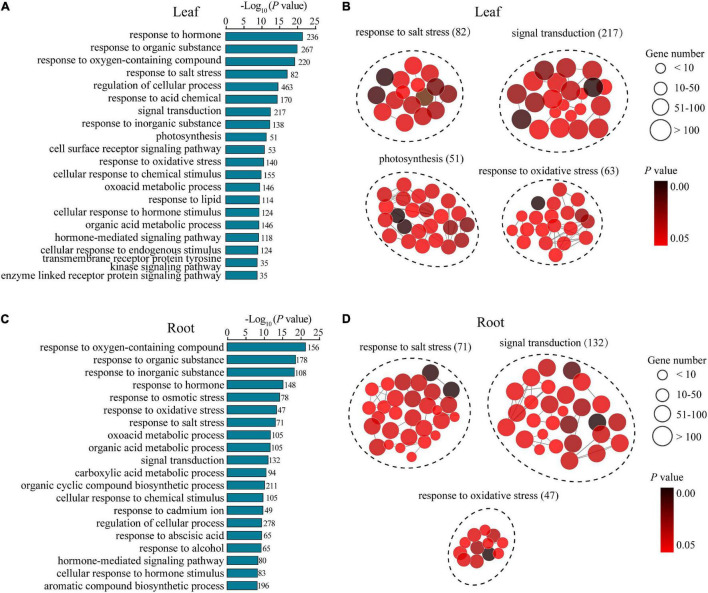
The Gene Ontology (GO) enrichment analysis of the differentially expressed genes (DEGs) in leaves and roots. **(A,B)** the GO terms of the DEGs generated from comparison R7SL vs. S4SL in leaves. **(C,D)** the GO terms of the DEGs generated from comparison R7SR vs. S4SR in leaves.

To understand the potential mechanisms underlying the distinguishing R7 and S4 in response to salt stress, a GO enrichment analysis of the DEGs in the comparisons R7SL vs. S4SL and R7SR vs. S4SR was performed, respectively. The results showed that multiple stress-associated GO terms were significantly enriched in both leaves and roots, such as response to an inorganic substance, response to salt stress, response to an organic substance, and response to the hormone (Figures 3A,C). Several GO terms related to oxidative stress, signal transduction, and metabolic process were also enriched in both leaves and roots. In addition, several GO terms, namely, photosynthesis, transmembrane receptor protein tyrosine kinase signaling pathway, and cell surface receptor signaling pathway were specifically enriched in leaves, whereas the terms including response to cadmium ion and response to osmotic stress were peculiarly enriched in roots (Figures 3B,D and Supplementary Table 5). These results suggested that salt stress induces the higher enrichment of specific stress-responsive genes in R7 than in S4.

### *Fraxinus velutina* R7 Leaves Enriched Hormonal and MAPK Signaling Pathways Under Salt Stress

**FIGURE 4:**
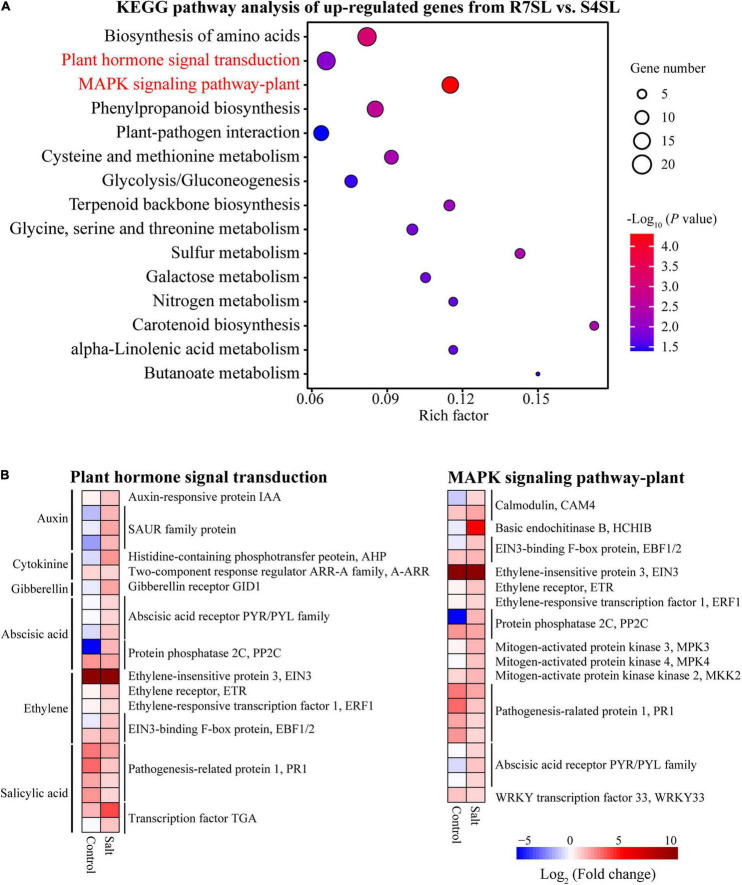
The Kyoto Encyclopedia of Genes and Genomes (KEGG) enrichment analysis of the differentially upregulated genes from leaves. **(A)** The top 15 enriched pathways (ranked by *P*-value) of upregulated genes in comparison R7SL vs. S4SL were showed. The size of dot represented the number of DEGs. From blue to red represented the *P*-value from low to high. **(B)** Heatmap showed the expression patterns of DEGs related to plant hormone signal transduction and MAPK signaling pathway pant. From blue to red represented the fold change in tolerant leaves compared to sensitive leaves from low to high.

To identify the signaling pathways involved in enhancing the salt tolerance of R7, the KEGG pathway enrichment analysis was performed using upregulated and downregulated DEGs from the comparison R7SL vs. S4SL. The results showed that the upregulated DEGs were mainly enriched in “plant hormone signal transduction” and “MAPK signaling pathway-plant” (Figure 4A and Supplementary Table 6), whereas the downregulated DEGs were enriched in “photosynthesis” and “photosynthesis-antenna proteins” (Supplementary Figure 4).

There were 23 DEGs involved in “plant hormone signal transduction,” namely, the signaling network of auxin (IAA and SAUR), cytokinin (AHP and A-ARR), gibberellin (GID1), abscisic acid (PYR/PYL and PP2C), ethylene (EIN3, ETR, ERF1, and EBF1/2), and salicylic acid (PR1and TGA). In addition, there were 21 DEGs involved in the “MAPK signaling pathway plant.” All these genes exhibited upregulation in the leaves of *F. velutina* R7 in comparison to the one in S4 under salt treatment (Figure 4B). The findings indicated that hormonal and MAPK signaling pathways are altered in the leaves of R7 after salt exposure.

### *Fraxinus velutina* R7 Roots Accumulated More Soluble Sugar and Proline Than S4 Under Salt Stress

**FIGURE 5:**
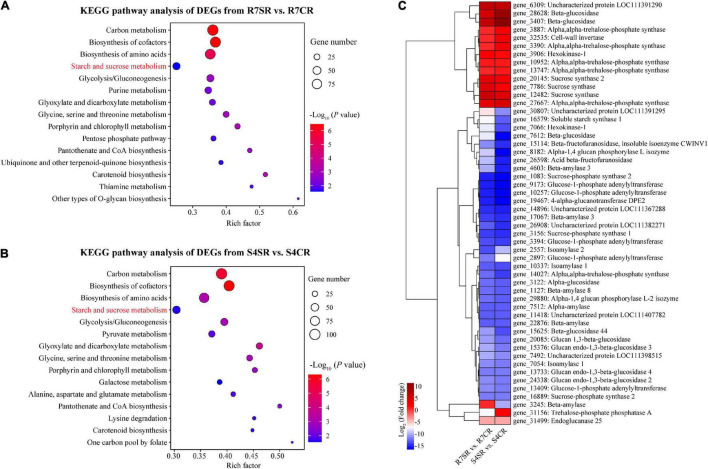
Functional enrichment analysis of the DEGs and measuring soluble sugar contents in *F. velutina* R7 and S4 roots. **(A,B)** The top 15 enriched KEGG pathways (ranked by *P*-value) for DEGs in response to salt stress in tolerant **(A)** and sensitive roots **(B)** were showed. **(C)** The expression patterns of DEGs associated with “starch and sucrose metabolism” pathway in R7 and S4 roots underlying salt conditions. Heatmap colors represented the fold change and from blue to red represented the value from low to high.

To explore the differences between R7 and S4 roots in response to salt stress, KEGG pathway enrichment analysis was performed based on the DEGs from comparisons R7SR vs. R7CR and S4SR vs. S4CR, respectively (Supplementary Table 7). The results showed that three pathways were overlapped between two comparisons included “carbon metabolism,” “biosynthesis of amino acids,” and “starch and sucrose metabolism” (Figures 5A,B).

Starch and sucrose metabolism can regulate osmotic adjustment *via* determining the contents of soluble sugars, playing critical roles in salt tolerance (Dahro et al., 2016; Wei et al., 2019). Thus, the expression patterns of DEGs in “starch and sucrose metabolism” in R7 and S4 roots under salt stress were further analyzed. The results showed that the expression patterns of several genes were different between R7 and S4 roots under salt stress (Figure 5C), which was consistent with the significantly elevated levels of soluble sugars in R7 roots than in S4 (Figure 1C), suggesting that starch and sucrose metabolism induced by salt stress in R7 and S4 roots is different.

**FIGURE 6:**
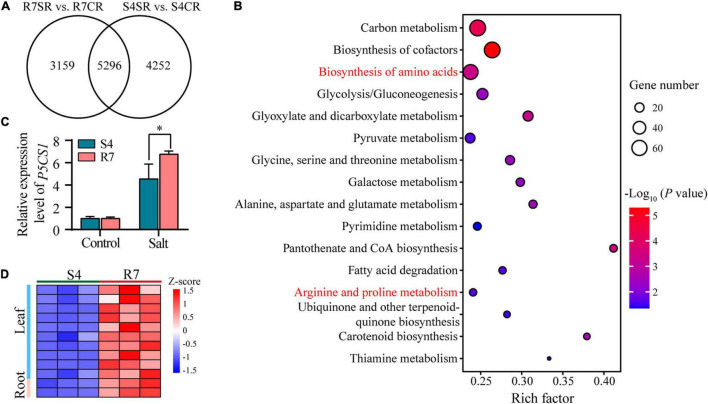
The KEGG pathway enrichment analysis of the shared DEGs between comparisons R7SR vs. R7CR and S4SR vs. S4CR and further analysis of proline metabolism. **(A)** Venn diagram of DEGs in response to salt stress in *F. velutina* R7 and S4 roots. **(B)** The significantly enriched pathways (*P* < 0.05). Size of dot represented the number of DEGs and from blue to red represented the *P*-value from low to high. **(C)** Detecting the relative expression levels of *P5CS1* in *F. velutina* R7 and S4 roots with or without salt treatment. **(D)** Relative expression levels of a peroxidase (POD) gene in R7 and S4 roots with or without salt treatment. Heatmap color represented z-score and from blue to red represented the value from low to high. *P < 0.05 represented significant differences.

To explore the common mechanisms in response to salt stress between R7 and S4 roots, an intersection analysis between the comparisons R7SR vs. R7CR and S4SR vs. S4CR was performed. The results identified 5,296 shared DEGs in *F. velutina* R7 and S4 roots under salt stress (Figure 6A). KEGG enrichment analysis of these genes revealed 16 significantly enriched pathways, such as “biosynthesis of amino acids” and “Arginine and proline metabolism” (Figure 6B and Supplementary Table 8), suggesting that accumulation of amino acids might improve the salt tolerance in both *F. velutina* R7 and S4 roots. Proline acting as an osmoprotectant plays an important role in improving salt tolerance in the plant (Liang et al., 2018; Rady et al., 2019). As a rate-limiting enzyme involved in proline biosynthesis (Turchetto-Zolet et al., 2009), a gene encoding delta-1-pyrroline-5-carboxylate synthase 1 (P5CS1) was differentially upregulated to higher levels in R7 roots than in S4 (Figure 6C), which was consistent with the significantly elevated levels of proline in R7 roots than in S4 (Figure 1D).

### *Fraxinus velutina* R7 Roots and Leaves Accumulated Less Reactive Oxygen Species Than S4

The increase in ROS levels leads to oxidative stress, posing detrimental effects on plant cells and tissues (Choudhury et al., 2017). Therefore, the DEGs involved in response to oxidative stress were analyzed further *via* dissecting the oxidative stress-related GO terms. Among the genes, 12 encoding peroxidases (PODs) were upregulated higher in R7 leaves (10 genes) and roots (2 genes) than in S4 (Figure 6D and Supplementary Table 4) under salt stress. Consistently, R7 leaves and roots presented lower levels of H_2_O_2_ than S4 under salt stress (Figures 1E–G). Collectively, these results suggested that R7 exhibits higher ROS scavenging capacity than S4 under salt stress.

### Several Transcription Factors Were Involved in Defense Response of *Fraxinus velutina* R7 Against Salt Stress

**FIGURE 7:**
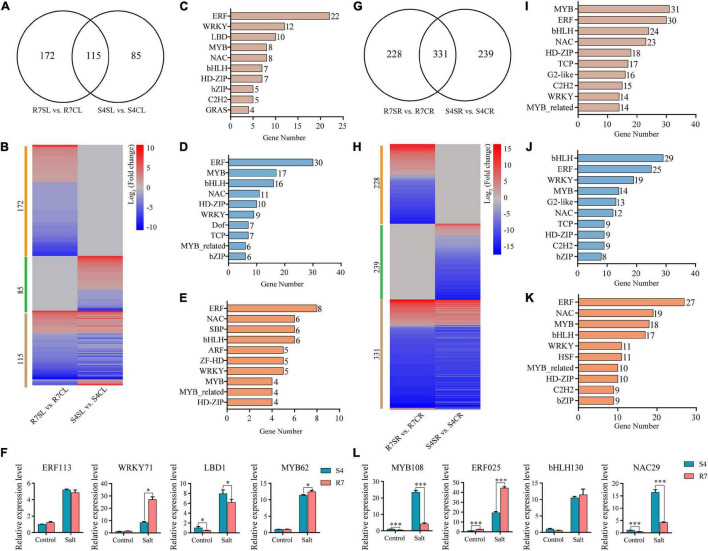
Analysis of the differentially expressed transcription factors (TFs) in R7 and S4. **(A,G)** Venn diagrams showed the distribution of salt-induced TFs in R7 and S4 leaves and roots. **(B,H)** Heatmap showed the fold change of salt-treated R7 and S4 roots and leaves compared to their corresponding non-treated samples. **(A–E, I–K)** The top 10 TF families (ranked by gene number) in specific and common TFs among different comparisons. **(F,L)** The relative expression levels of the shared differentially expressed TFs in leaves and roots. *P < 0.05 and ^***^P < 0.001 represented significant differences.

Transcription factors (TFs) act as the master switches in regulating multiple downstream target genes, thus playing crucial roles in various biological processes including salt stress response. By dissecting the RNA sequencing data, multiple TF genes were significantly altered between R7 and S4 under salt stress (Figure 7 and Supplementary Tables 9, 10). Among the significantly expressed TFs, 172 and 85 TFs were specifically expressed in R7SL vs. R7CL and S4SL vs. S4CL, respectively, and 115 were overlapped in both R7 and S4 (Figures 7A,B). Of the shared TFs, ERF, WRKY, LBD, and MYB were the most represented TF families induced by salt stress (Figure 7C).

In leaves under salt stress, 30 genes were encoding ERF, 17 encoding MYB, 16 encoding bHLH, and 11 encoding NAC prominently expressed in R7 (Figure 7D), whereas there were 8 encoding ERF, 6 encoding NAC, 6 encoding SBP, and 6 encoding bHLH prominently expressed in S4 (Figure 7E). In root tissues, bHLH, ERF, WRKY, and MYB TF families were prominently induced by salt stress in both R7 and S4 (Figures 7H,I). There were 29 genes encoding bHLH, 25 encoding ERF, and 19 encoding WRKY preferentially induced in R7 (Figure 7J), whereas 27 genes were encoding ERF, 19 encoding NAC, and 18 encoding MYB preferentially induced in S4 (Figure 7K).

To further confirm the roles of these TFs in regulating the stress response, 8 TF genes, namely, *ERF113*, *WRKY71*, *LBD1*, and *MYB62* in leaves and *MYB108*, *ERF025*, *bHLH130*, and *NAC29* in roots were chosen to perform qRT-PCR analysis. The results showed that both *WRKY71* and *MYB62* were significantly upregulated higher in R7 leaf than in S4, while *LBD1* was downregulated in R7 compared with S4 (Figure 7F). In addition, *MYB108* and *NAC29* were found downregulated in R7 root than in S4, whereas *ERF025* was significantly upregulated in R7 in comparison to S4 (Figure 7L). These results suggested that the stress-responsive TFs, such as *WRKY71*, *MYB62*, and *ERF025* are the important contributors to enhancing salt tolerance in R7.

## Discussion

In this study, to explore the mechanisms underlying salt tolerance of *F. velutina*, a comparative transcriptome analysis was performed on the leaf and root of two contrasting materials, *F. velutina* R7 (salt-tolerant) and S4 (salt-sensitive) clone exposed to salt stress. The results showed that the high salt tolerance of R7 is mainly attributed to the enrichment of stress-responsive genes. The stress-responsive genes in the R7 leaf were associated with plant hormone signaling and MAPK signaling pathways, whereas the genes in the root were involved in proline biosynthesis and starch and sucrose metabolism. The genes encoding POD were upregulated after salt exposure, resulting in high ROS scavenging capacity in R7.

Plant hormones are the important contributors of plant growth and developmental processes and play crucial roles in biotic and abiotic stress responses (Wang et al., 2013; Xia et al., 2015; Kaleem et al., 2018). In this study, the pathway “plant hormone signal transduction” was significantly enriched in the R7 leaf, which included 23 DEGs related to auxin, cytokinin, gibberellin, abscisic acid, ethylene, and salicylic acid. Our results were consistent with the previous studies showed that these hormones play irreplaceable roles in the response to salt stress in plants (Zheng et al., 2018; Feng et al., 2019; Gao et al., 2021; Huang et al., 2021; Saini et al., 2021; Zhu et al., 2021). For instance, salt stress can induce ABA signaling and activate an ABA-dependent responsive complex to cope with saline stress in plants (Umezawa et al., 2009; Cai et al., 2017; Soma et al., 2017; Lin et al., 2021). In this study, 5 DEGs related to ABA signal transduction were significantly affected under salt stress, such the significantly upregulated genes encoding protein phosphatase (PP2C), which has been confirmed that the upregulation of *PP2C* can lead to enhanced salt tolerance in *Arabidopsis* and maize (Liu et al., 2012). These findings suggested that the activation of hormone signaling improves salt tolerance in plants. In addition, we found that *PYR1/PYL/RCAR* ABA receptor genes and *PP2C* were the top salt responsive genes, suggesting that these genes are the candidate genes for developing salinity resilience through genetic engineering in the future.

The stress-responsive genes in the R7 leaf were also involved in the MAPK signaling pathway, which is documented to modulate plant tolerance to various abiotic stress, such as salt stress (De Zelicourt et al., 2016). The pathway included multiple genes that respond to salt stress, such as calmodulin (*CAM4*), mitogen-activated protein kinase 3 (*MPK3*), and WRKY transcription factor 33 (*WRKY33*). The overexpression of *MsCML46* in tobacco can lead to enhanced tolerance to multiple stresses, namely, drought, freezing, and salt stress (Du et al., 2021). In potatoes, it is confirmed that the overexpression of *MPK3* can enhance osmosis and salinity tolerances by modulating the antioxidant system and proline biosynthesis (Zhu et al., 2020). In *Arabidopsis*, *WRKY33* leads to enhanced salt tolerance by modulating *CYP94B1* expression (Krishnamurthy et al., 2020). These results indicated that the genes involved in the MAPK signaling pathway are important contributors to high salt tolerance in plants, especially *CAM4*, *MPK3*, and *WRKY33* which are the important candidate genes for future salt-tolerant plants breeding through genetic engineering.

Under salt stress conditions, the compatible osmolytes soluble sugars and proline are critical for adjusting osmotic potential induced by excessive salt stress (Zhu, 2016; Liang et al., 2018). Increasing evidence indicated that the accumulation of soluble sugars and proline is associated with enhanced stress tolerance in plant cells (Mansour and Ali, 2017; Dai et al., 2018; Rady et al., 2019). Proline content, antioxidant activities, and potassium content are influenced in response to abiotic stresses for controlling membrane stability and mitigating the toxicity of sodium (Heidari et al., 2021; Musavizadeh et al., 2021). In our study, a gene encoding P5CS1, a rate-limiting enzyme involved in proline biosynthesis (Turchetto-Zolet et al., 2009), was differentially upregulated to higher levels in R7 roots than in S4. Meanwhile, significantly elevated levels of proline were also found in R7 roots. In addition, the contents of soluble sugars were determined and we found R7 roots exhibited higher levels of soluble sugars than S4. These results suggested that the increased levels in soluble sugars and proline lead to enhanced salt tolerance and that the salt stress-induced *P5CS1* is a crucial candidate for salt-tolerant engineering in *F. velutina*.

Salt stress causes oxidative stress by promoting the generation of ROS in the plant (Zhu, 2016). It is documented that plants can alleviate ROS-caused damages by activating several enzymatic and non-enzymatic pathways involved in the antioxidant system (Choudhury et al., 2017). Previous studies have revealed that the improved ROS scavenging capacity can enhance plant tolerance to several stresses (Gill and Tuteja, 2010; Zhang et al., 2015; Wei et al., 2019). In our study, 12 genes encoding POD were upregulated to higher in R7 compared with S4 under salt stress. Consistently, lower levels of H_2_O_2_ and fewer cell damages were found in R7 than in S4.

## Conclusion

**FIGURE 8:**
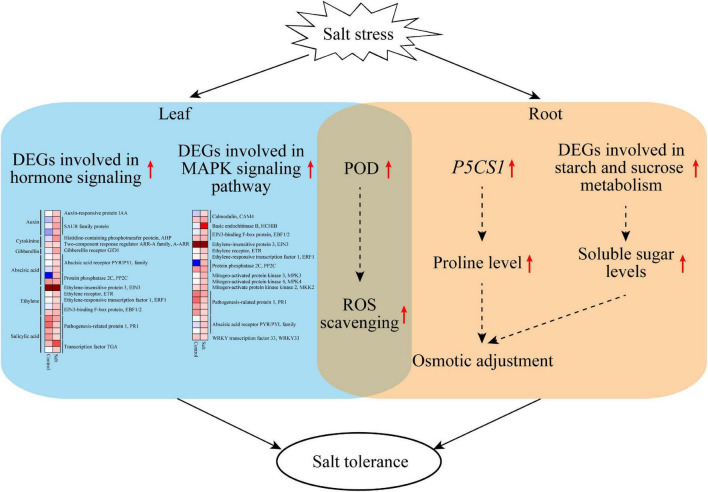
A model proposed potentially to explain the high salt tolerance of *F. velutina* R7. Under salt stress, R7 activates multifaceted defense machines in the roots and leaves to enhance salt tolerance. In the R7 root, salt stress induces the upregulation of *P5CS1* and the key genes involved in starch and sucrose metabolism, therefore increasing osmotic adjustment. In the R7 leaf, salt stress induces the upregulation of important genes involved in plant hormone signaling (especially *PYR1/PYL/RCAR* ABA receptor genes and *PP2C*) and MAPK signaling pathways (especially *CAM4*, *MPK3*, and *WRKY33*). In addition, salt stress induces the upregulation of *POD* genes in both roots and leaves, so enhancing ROS scavenging capacity.

This study revealed that *F. velutina* R7 presents higher salt tolerance than S4. The findings reported here allow us to propose a potential mechanism to explain the high salt tolerance of R7 (Figure 8). Under salt stress, R7 activates multifaceted defense machines in the roots and leaves to enhance salt tolerance. In the R7 root, salt stress induces the upregulation of *P5CS1* and the key genes involved in starch and sucrose metabolism, therefore increasing osmotic adjustment. In the R7 leaf, salt stress induces the upregulation of important genes involved in plant hormone signaling (especially *PYR1/PYL/RCAR* ABA receptor genes and *PP2C*) and MAPK signaling pathways (especially *CAM4*, *MPK3*, and *WRKY33*). In addition, salt stress induces the upregulation of *POD* genes in both roots and leaves, enhancing ROS scavenging capacity. Collectively, our findings revealed the crucial defense pathways underlying the high salt tolerance of R7, thus providing insights into the salt-tolerant *F. velutina* breeding.

## Data Availability Statement

The datasets presented in this study can be found in online repositories. The names of the repository/repositories and accession number(s) can be found in the article/Supplementary Material. The RNA-Seq data presented in this study can be available at the Sequence Read Archive under accession numbers PRJNA791967.

## Author Contributions

KY and DW conceptualized the research program. JL, XM, and LY finished the analysis of this study and wrote the manuscript. QL, HF, and CW conducted the RNA sequencing data analysis. YD and ZC designed the qRT-PCR experiment and finished the operation. RZ, YB, and WH planted the material and finished the physiology analysis. JL, KY, and DW revised the manuscript. All authors discussed the results, commented on the manuscript, and approved the submitted version.

## Conflict of Interest

The authors declare that the research was conducted in the absence of any commercial or financial relationships that could be construed as a potential conflict of interest.

## Publisher’s Note

All claims expressed in this article are solely those of the authors and do not necessarily represent those of their affiliated organizations, or those of the publisher, the editors and the reviewers. Any product that may be evaluated in this article, or claim that may be made by its manufacturer, is not guaranteed or endorsed by the publisher.

## Footnotes

1
http://ashgenome.org/

## Supplementary Material

Supplementary Figure 1 All the samples were hierarchical clustered by calculating correlation coefficient (γ^2^) between each sample. From blue to red represented the value of γ^2^ from low to high.

Supplementary Figure 2 Identification of the differentially expressed genes (DEGs) in leaves. **(A)** Heatmap showed the expression patterns of DEGs in comparisons S4SL vs. S4CL, R7CL vs. S4CL, R7SL vs. S4SL, and R7SL vs. R7CL based on Z-score. The color represented the Z-score. From blue to red represented the Z-score from low to high. **(B)** Venn diagram showed the distribution of upregulated and downregulated DEGs among different comparisons, respectively.

Supplementary Figure 3 Identification of the differentially expressed genes (DEGs) in roots. **(A)** Heatmap showed the expression patterns of DEGs in comparisons S4SR vs. S4CR, R7CR vs. S4CR, R7SR vs. S4SR, and R7SR vs. R7CR based on Z-score. The color represented the Z-score. From blue to red represented the Z-score from low to high. **(B)** Venn diagram showed the distribution of upregulated and downregulated DEGs among different comparisons, respectively.

Supplementary Figure 4 The Kyoto Encyclopedia of Genes and Genomes (KEGG) pathway analysis of downregulated genes in comparison R7SL vs. S4SL.
