# Supplementary figures and images for: Comparative Transcriptome Analysis Unravels Defense Pathways of Fraxinus velutina Torr Against Salt Stress
Source: Front Plant Sci. 2022 Mar 4;13:842726. doi: 10.3389/fpls.2022.842726 (PMC8931533; doi:10.3389/fpls.2022.842726)

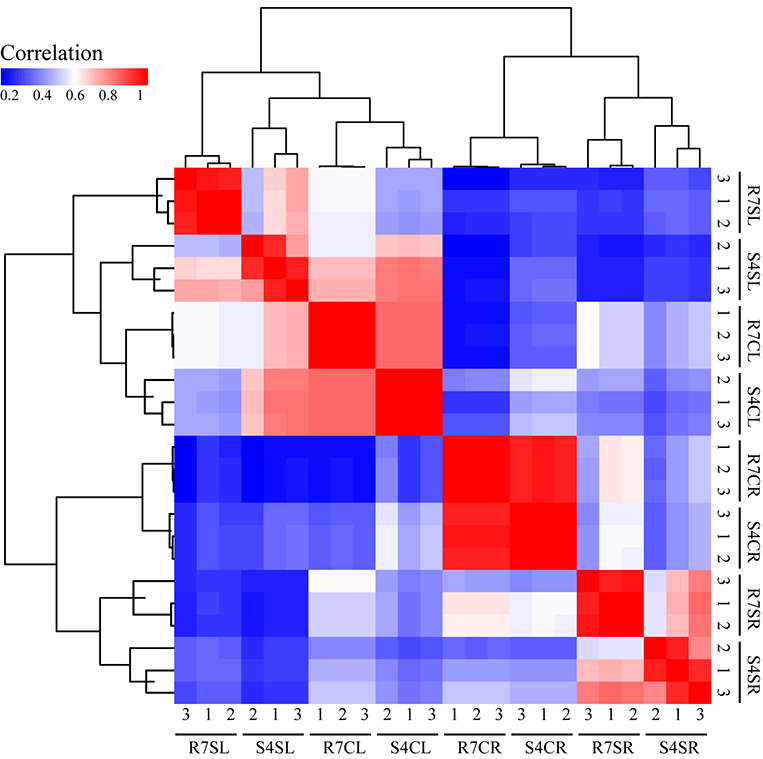

Supplement: Supplementary Figure 1 — All the samples were hierarchical clustered by calculating correlation coefficient (γ2) between each sample. From blue to red represented the value of γ2 from low to high. [file Image_1.TIF]

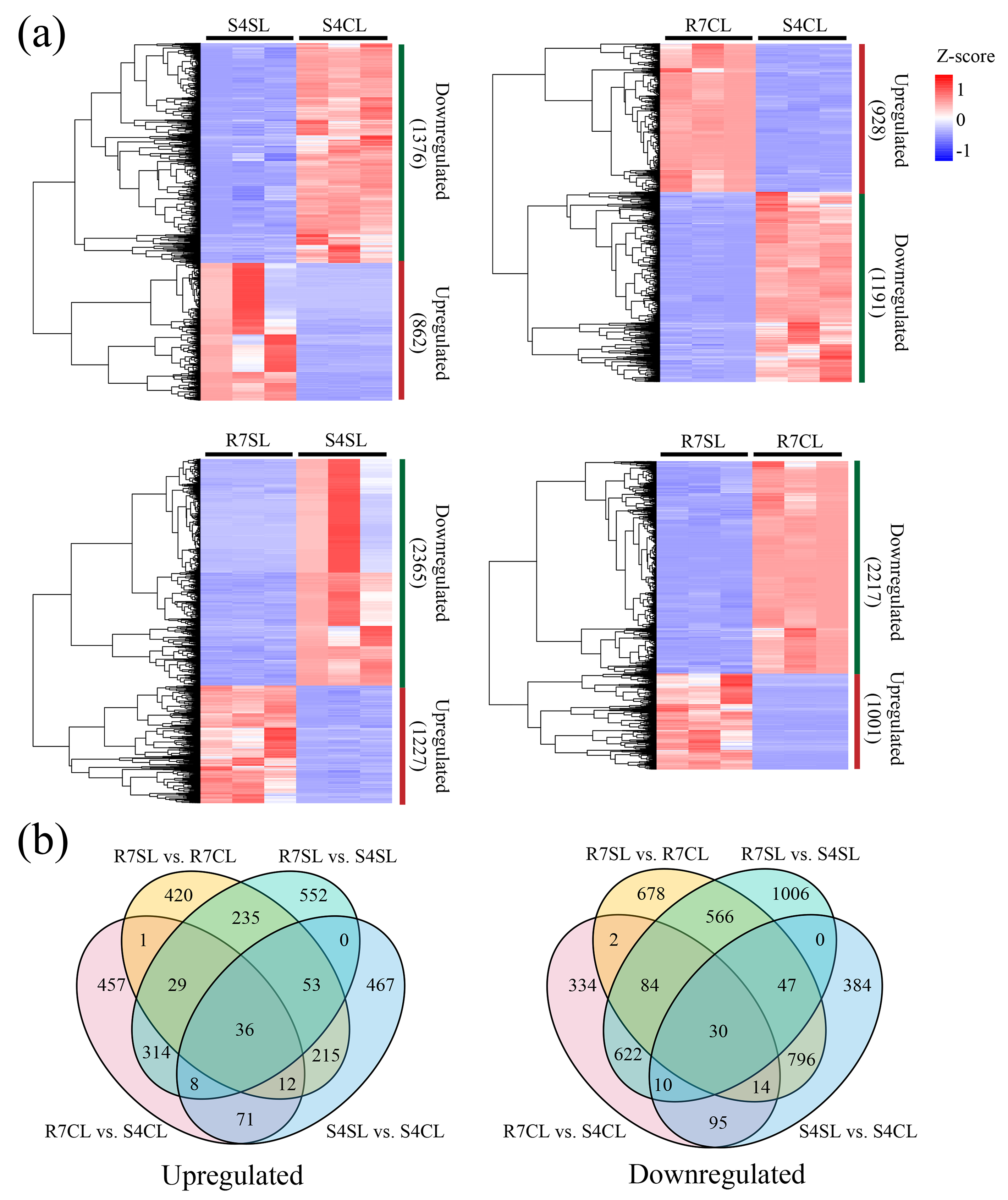

Supplement: Supplementary Figure 2 — Identification of the differentially expressed genes (DEGs) in leaves. (A) Heatmap showed the expression patterns of DEGs in comparisons S4SL vs. S4CL, R7CL vs. S4CL, R7SL vs. S4SL, and R7SL vs. R7CL based on Z-score. The color represented the Z-score. From blue to red represented the Z-score from low to high. (B) Venn diagram showed the distribution of upregulated and downregulated DEGs among different comparisons, respectively. [file Image_2.TIF]

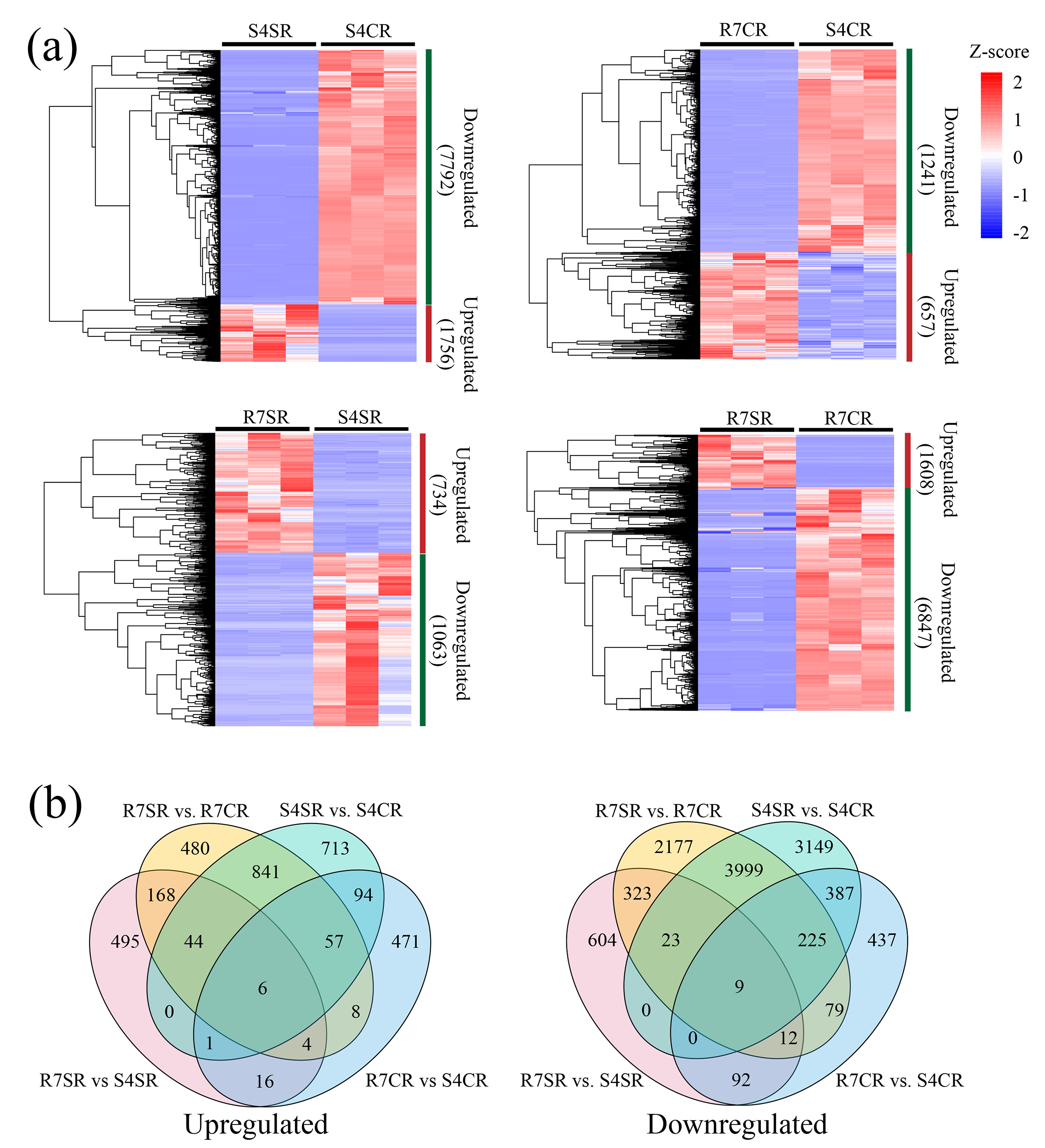

Supplement: Supplementary Figure 3 — Identification of the differentially expressed genes (DEGs) in roots. (A) Heatmap showed the expression patterns of DEGs in comparisons S4SR vs. S4CR, R7CR vs. S4CR, R7SR vs. S4SR, and R7SR vs. R7CR based on Z-score. The color represented the Z-score. From blue to red represented the Z-score from low to high. (B) Venn diagram showed the distribution of upregulated and downregulated DEGs among different comparisons, respectively. [file Image_3.TIF]

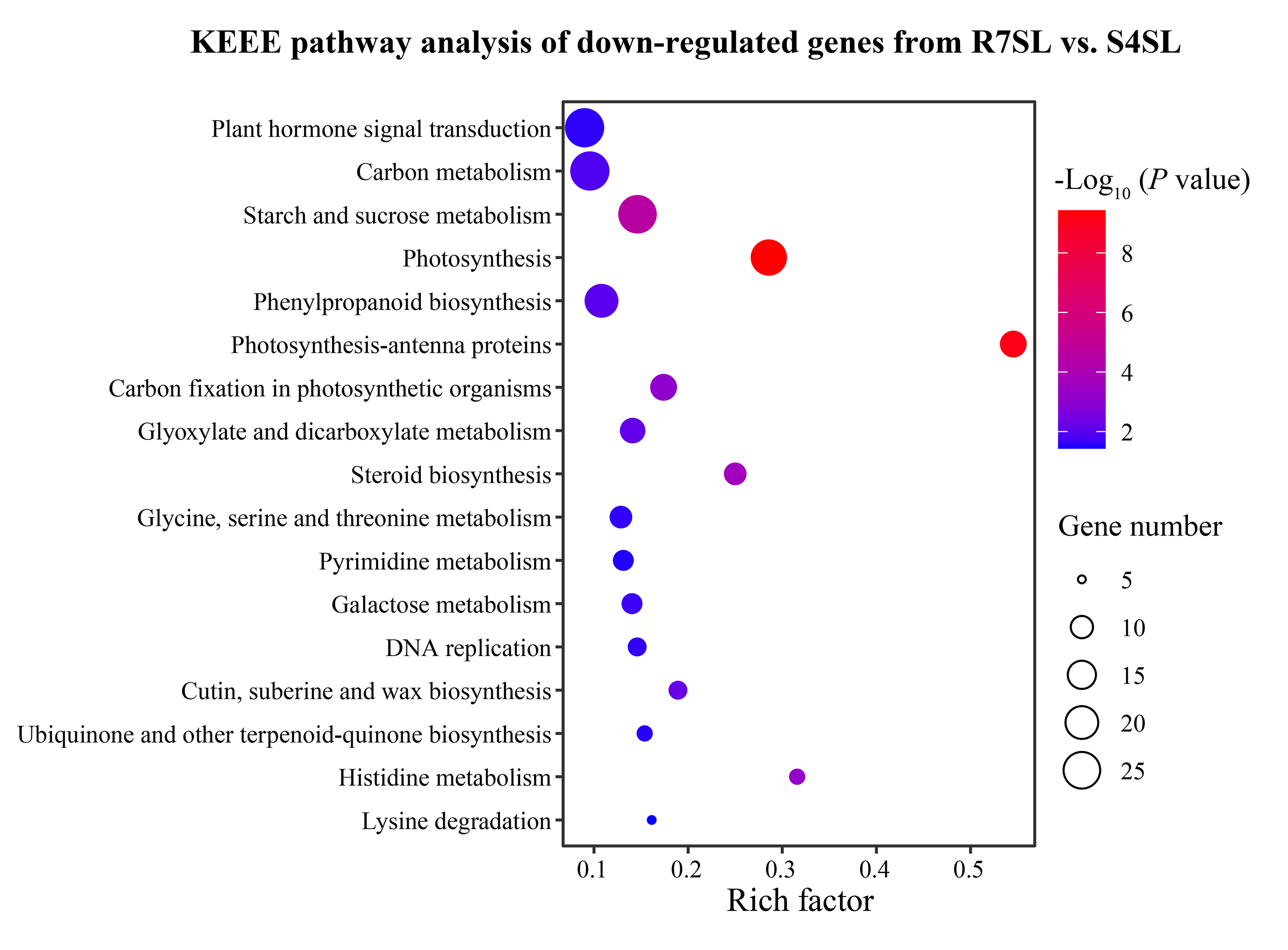

Supplement: Supplementary Figure 4 — The Kyoto Encyclopedia of Genes and Genomes (KEGG) pathway analysis of downregulated genes in comparison R7SL vs. S4SL. [file Image_4.TIF]
